# miR-320a is an independent prognostic biomarker for invasive breast cancer

**DOI:** 10.3892/ol.2014.2298

**Published:** 2014-06-30

**Authors:** HAIPING YANG, JUAN YU, LEI WANG, DI DING, LEI ZHANG, CHENGYU CHU, QI CHEN, ZUDE XU, QIANG ZOU, XIUPING LIU

**Affiliations:** 1 Department of Pathology, School of Basic Medical Sciences, Fudan University, Shanghai 200032, P.R. China; 2 Department of Surgery, Huashan Hospital, Fudan University, Shanghai 200040, P.R. China; 3 Department of Pathology, The Fifth People’s Hospital of Shanghai, Fudan University, Shanghai 200240, P.R. China

**Keywords:** miR-320a, breast cancer, prognosis, biomarker, chromogenic *in situ* hybridization

## Abstract

Breast cancer is one of the most common malignancies worldwide and is the second leading cause of cancer-related mortality among females. miRNAs are a class of small noncoding RNAs that are aberrantly expressed in human cancers. Due to their small size and stability, miRNAs have the potential to be efficacious clinical targets. MicroRNA-320a (miR-320a) has been shown to be dysregulated in multiple malignancies. In the present study, the expression levels of miR-320a were investigated in 15 paraffin-embedded *in situ* breast carcinoma and 130 invasive breast cancer tissues, and the prognostic value for breast cancer patients was assessed. Chromogenic *in situ* hybridization revealed that 60/130 (46%) invasive breast cancer tissues exhibited high expression levels of miR-320a (staining index score of ≥4). Furthermore, miR-320a staining was found to significantly correlate with tumor size (P=0.046), clinical stage (P<0.001), lymph node metastasis (P<0.001) and distant metastasis (P=0.006). In addition, patients exhibiting low miR-320a expression levels had shorter overall survival times (P<0.001). Univariate and multivariate analyses revealed that miR-320a was an independent prognostic biomarker for invasive breast cancer (hazard ratio, 0.221; 95% confidence interval, 0.050–0.979; P=0.047). Receiver operator characteristic curves revealed that the prognostic value of miR-320a was enhanced when compared with the widely used prognostic biomarkers (estrogen receptor, progesterone receptor and human epidermal growth factor-2) in invasive breast cancer. The results of the present study suggest that miR-320a presents a potential biomarker for the prognosis of invasive breast cancer, and dysregulation of miR-320a may be involved in invasive breast cancer progression.

## Introduction

Breast cancer is one of the most common types of malignant tumor worldwide and is the second leading cause of cancer-related mortality among females (1). Genetic alterations, environmental toxins, hormones, diet and stress are the predominant causes of breast cancer. Although improved early detection and effective treatment may help to prolong the survival times of breast cancer patients, numerous patients succumb to the disease as a result of invasion and metastasis (2). Therefore, it is important to identify effective predictive biomarkers to provide more accurate diagnoses and to develop novel therapeutic strategies.

MicroRNAs (miRNAs) are a class of short (18–24 nucleotides), non-protein-coding RNAs that bind to the 3′ untranslated regions (3′ UTRs) of target mRNAs to regulate gene expression by inhibiting the translation of target mRNAs or by promoting transcript degradation (3–5). A number of studies have found that miRNAs are important in a number of biological processes, including cell differentiation, proliferation, apoptosis and metabolism (6). miRNAs are also involved in the process of cancer development, progression and metastasis, exhibiting oncogenic or tumor suppressor functions (7). For example, miR-34a expression is reduced in neuroblastoma and acts as a tumor suppressor (8); however, miR-155 is overexpressed in chronic lymphocytic leukemia and acts as an oncogene (9).

Previous studies have revealed that miR-320a exhibits abnormal expression levels in multiple malignancies and is involved in the formation, progression and metastasis of cancer. Sun *et al* (10) reported that miR-320a suppressed human colon cancer cell proliferation by directly targeting β-catenin. miR-320a also inhibits tumor invasion by targeting neuropilin-1 and is associated with liver metastasis in colorectal cancer (11). However, Xu *et al* (12) reported that miR-320a was upregulated two- to 14-fold in prostate cancer cells, and may exhibit an oncogenic function in prostate cancer. Recently, Xu *et al* (13) revealed that miR-320a was a potentially valuable biomarker for diagnosing older females with gastric cancer. However, few studies have investigated the clinicopathological value and prognostic significance of miR-320a expression in breast cancer.

In the present study, the miR-320a expression levels in 15 *in situ* breast carcinoma and 130 invasive breast cancer samples were examined using chromogenic *in situ* hybridization. The results demonstrated that miR-320a was downregulated in invasive breast cancer. Furthermore, low miR-320a expression was found to be associated with invasive breast cancer progression and predicts poor patient prognosis.

## Materials and methods

### Patients and tissue samples

Paraffin-embedded invasive breast cancer samples from 130 patients (mean age, 55.7 years; range, 34–87 years) were obtained between January 1999 and December 2002. A total of 15 paraffin-embedded *in situ* breast carcinoma tissues were collected in 2011. All tissues were obtained from Huashan Hospital of Fudan University (Shanghai, China). None of the 130 invasive breast cancer patients received chemotherapy or radiation therapy prior to surgery. The study was approved by the ethical committee of Huashan Hospital, Fudan University (Shanghai, China), and all patients provided written informed consent.

A total of 102 (78.5%) invasive ductal carcinomas, 15 (11.5%) lobular carcinomas, eight (6.2%) medullary carcinomas and five (3.8%) mucinous adenocarcinomas were identified among the 130 invasive breast cancer samples. The clinical tumor lymph node metastasis (TNM) stage of each cancer was based on the World Health Organization guidelines (14), and the histological grade was classified according to Scarff-Bloom-Richardson grading (15). All 130 cases were followed-up after surgery, and the final date of follow-up was December 31, 2008. The mean duration of follow-up was 77.5 months. The overall survival rates were calculated from the date of resection to the follow-up deadline or date of mortality. The clinicopathological characteristics of the patients and follow-up data are shown in Table I.

### Chromogenic in situ hybridization (CISH)

Chromogenic *in situ* hybridization (CISH) was used to detect miR-320a expression levels in 15 paraffin-embedded *in situ* breast carcinoma and 130 invasive breast cancer samples. Briefly, following dewaxing in xylene and rehydrating in graded alcohol, the slides were digested with pepsin. The slides were then prehybridized in a prehybridization solution at 54°C for 2 h. Following prehybridization, 5′digoxin-conjugated locked nucleic acid probes for miR-320a, U6 (positive control) and scrambled RNA (negative control) (all Exiqon, Copenhagen, Denmark) were used for hybridization at 54°C for 16–20 h. Following washing with Tris-buffered saline, the slides were incubated with a sheep polyclonal anti-digoxin antibody (Roche Diagnostics GmbH, Mannheim, Germany). Next, the slides were stained with nitro blue tetrazolium/5-bromo-4-chloro-3-indolyl-phosphate. Methyl green was used to counterstain the nuclei. Positive results appeared blue in the cytoplasm and nuclei.

The slides were scored independently by two pathologists, and positive nuclear and cytoplasmic miR-320a expression was detected. The proportion of positively stained tumor cells and the staining intensity were evaluated over 10 visual fields (magnification, ×40; BX-51, Olympus America Inc., Melville, NY, USA). For statistical analysis, with reference to Tang *et al* (16), total staining of miR-320a was analyzed based on the proportion of positively stained tumor cells identified, and the following four scores were used: 0, no positive tumor cells; 1, <10% positive tumor cells; 2, 10–50% positive tumor cells; and 3, >50% positive tumor cells. The staining intensity was also analyzed according to four scores: 0, no staining; 1, light blue/weak staining; 2, blue/moderate staining; and 3, dark blue/strong staining. The staining index (SI) was calculated using the following formula: SI = staining intensity × proportion of positively stained tumor cells. Using the aforementioned method, the expression of miR-320a was scored as 0, 1, 2, 3, 4, 6 or 9. An SI score of 4 was selected as a cut-off value based on a measurement of heterogeneity with the log-rank test statistic with respect to overall survival (16,17), and the expression levels of miR-320a were defined as high (SI≥4) or low (SI<4).

### Statistical analysis

Statistical analyses were performed using SPSS, version 19.0 (SPSS, Inc., Chicago, IL, USA). The χ^2^ test and Fisher’s exact test were used to analyze the association between miR-320a expression and clinicopathological features. Survival curves were generated using the Kaplan-Meier method and compared using the log-rank test. Variables with P<0.05 in the univariate analysis were entered into the Cox regression analysis, and the multivariate analysis used the Cox proportional-hazards model. Receiver operating characteristic (ROC) curves were generated using MedCalc, version 10.4.7.0 (MedCalc, Mariakerke, Belgium). P<0.05 was considered to indicate a statistically significant difference.

## Results

### miR-320a expression in invasive breast cancer and in situ breast carcinoma

**Figure 1:**
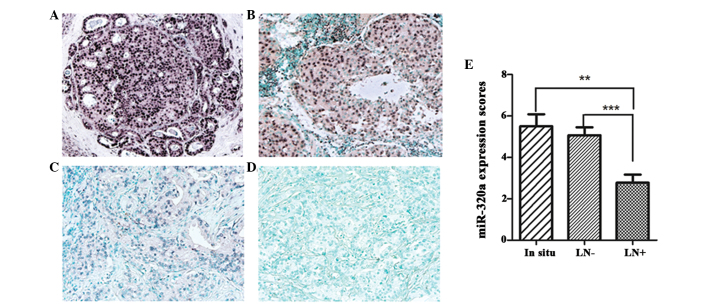
miR-320a is underexpressed in invasive breast cancer tissues. Representative images (magnification, ×200) of chromogenic *in situ* hybridization for miR-320a in (A) *in situ* breast carcinoma, (B) invasive breast cancer without lymph node metastasis and (C) invasive breast cancer with lymph node metastasis. (D) Scramble RNA was used as a negative control. (E) miR-320a expression scores were compared between invasive breast cancer tissues and *in situ* breast carcinoma tissues (P<0.01). ^**^P<0.01, *in situ* vs. LN^+^; and ^***^P<0.001, LN^+^ vs. LN^−^. In situ, *in situ* breast carcinoma; LN−, invasive breast cancer tissues without lymph node metastasis; LN+, invasive breast cancer tissues with lymph node metastasis. miR-320a, microRNA-320a.

CISH was used to detect miR-320a expression in 15 *in situ* breast carcinoma and 13 invasive breast cancer tissues. miR-320a expression was observed in the nuclei and cytoplasm, predominately in luminal epithelial cells (Fig. 1). High miR-320a expression (SI≥4) was detected in 12/15 (80%) *in situ* breast carcinoma and 60/130 (46%) invasive breast cancer samples. In addition, levels of miR-320a expression in invasive breast cancer with lymph node metastasis were found to be significantly lower than levels for breast cancer without lymph node metastasis and *in situ* breast carcinoma (P<0.01; Fig. 1E). However, no significant differences were identified between *in situ* breast carcinoma and invasive breast cancer without lymph node metastasis. These results suggest that downregulated miR-320a expression may be involved in cancer progression.

### Correlation between miR-320a expression and clinicopathological characteristics in invasive breast cancer

To further evaluate whether low miR-320a expression was associated with the progression of breast cancer, we analyzed the correlation between miR-320a expression levels and the clinicopathological characteristics of 130 invasive breast cancer patients (Table II). It was found that miR-320a was downregulated in patients with a larger tumor size (P=0.046), more advanced clinical staging (P<0.001) and increased lymph node metastases (P<0.001), as well as the presence of distant metastasis (P=0.006). However, no significant differences were identified between the expression levels of miR-320a and age (P=0.164), histological grade (P=0.745), menopause (P=0.697), human epidermal growth factor-2 (HER-2) expression (P=0.290), estrogen receptor (ER) status (P=0.684) or progesterone receptor (PR) status (P=0.352).

### Low expression levels of miR-320a correlate with poor prognosis in 130 invasive breast cancer patients

**Figure 2:**
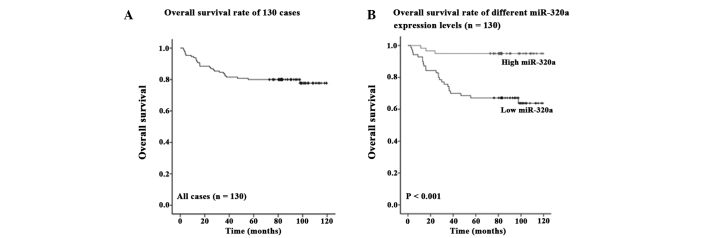
Low miR-320a levels are correlated with poor survival in invasive breast cancer patients. (A) The overall survival rate of 130 invasive breast cancer patients. (B) Kaplan-Meier survival curves and log-rank test showing the association between miR-320 expression levels and overall survival rate in 130 invasive breast cancer patients. miR-320a, microRNA-320a.

The prognostic value of miR-320a expression levels was evaluated in 130 invasive breast cancer patients using Kaplan-Meier analysis and the log-rank test. Among the 130 invasive breast cancers patients, 57/60 (95%) patients with high miR-320a expression survived, whereas 46/70 (66%) patients with low miR-320a expression survived. The overall survival rate of the 130 invasive breast cancer patients was 79% (Fig. 2A), and the overall survival rate of invasive breast cancer patients with low miR-320a expression levels was significantly shorter than that in patients with high miR-320a expression (P<0.001; Fig. 2B).

Using univariate survival analysis, the clinical TNM stage (P=0.010), menopause (P=0.020), miR-320a expression level (P=0.015) and distant metastasis (P=0.001) were found to be significantly associated with prognosis, however, no significant differences were identified between prognosis and age (P=0.587), lymph node metastasis (P=0.076), chemotherapy (P=0.900), tumor size (P=0.230), histological grade (P=0.977), ER expression (P=0.802), PR expression (P=0.445) or HER-2 expression (P=0.650) (Table III). Multivariate analyses were then used to determine whether miR-320a expression levels were an independent prognostic predictor of clinical outcomes. The results revealed that a decrease in miR-320a expression [hazard ratio (HR)=0.221; 95% confidence interval (CI), 0.050–0.979; P=0.047] and clinical TNM stage (HR, 4.434; 95% CI, 2.308–8.522; P<0.001) (Table III) showed significant prognostic effects on overall survival. Thus, these results indicated that miR-320a expression levels were significantly associated with invasive breast cancer patient prognosis.

### miR-320a is an indicator for predicting the prognosis of advanced-stage invasive breast cancer patients

**Figure 3:**
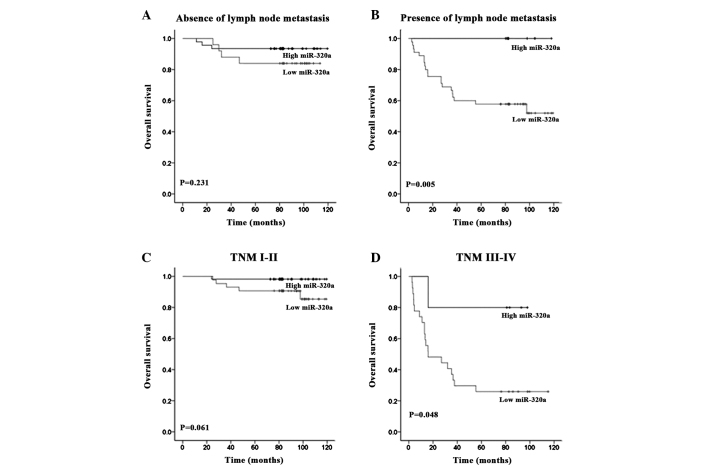
miR-320a shows improved prognostic value in advanced-stage breast cancer patients. Kaplan-Meier analysis and log-rank test showing the overall survival of 130 invasive breast cancer patients with low and high miR-320a levels categorized according to (A and B) lymph node status and (C and D) clinical TNM stage. miR-320a, microRNA-320a; TNM, tumor lymph node metastasis.

The prognostic value of miR-320a expression in selective patient subgroups classified by clinical TNM stage and lymph node status was evaluated. Low miR-320a expression was found to significantly correlate with poor survival in patients with clinical stage III–IV (P=0.048; Fig. 3D) and lymph node metastasis (P=0.005; Fig. 3B). However, no significant differences between high and low miR-320a expression groups were identified between invasive breast cancer patients with clinical stage I–II (P=0.061; Fig. 3C) and those without lymph node metastasis (P=0.231; Fig. 3A). These results indicate that miR-320a may present an improved prognostic biomarker for advanced-stage invasive breast cancer patients.

### miR-320a more effectively predicts invasive breast cancer prognosis when compared with commonly used clinicopathological prognostic biomarkers

**Figure 4:**
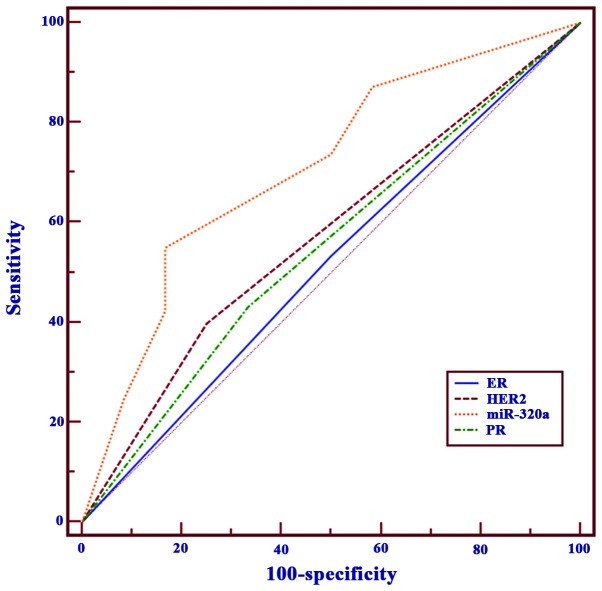
Comparison of miR-320a with commonly used prognostic parameters using ROC curves. The ROC areas under the curve for miR-320a, ER, PR and HER-2 were 0.710, 0.517, 0.549 and 0.574, respectively. miR-320a, microRNA-320a; ROC, receiver operating characteristic; ER, estrogen receptor; PR, progesterone receptor; HER-2, human epidermal growth factor.

ROC curves were used to compare the sensitivity and specificity of miR-320a expression with commonly used clinicopathological prognostic biomarkers (ER, PR and HER-2). The survival state of patients was used as the classification variable, whereas ER, PR, HER-2 (negative/positive) and the expression scores of miR-320a were used as the test variables. An area under the ROC curve of 0.7–0.9% was considered to present improved discrimination, whereas an ROC value of 0.5% indicated no discrimination (18). The ROC areas for miR-320a, ER, PR, and HER-2 were 0.710, 0.517, 0.549, and 0.574%, respectively. This result revealed that compared with routinely applied clinical prognostic biomarkers, miR-320a alone is a more reliable predictor for invasive breast cancer prognosis (Fig. 4).

## Discussion

miRNA profiles have been established for numerous solid and hematologic malignancies (19). In particular, miR-320a has been reported to be important in a number of cancer types (20–22); however, the association between clinicopathological characteristics and survival in breast cancer remains unclear. In the present study, miR-320a expression in 15 paraffin-embedded *in situ* breast carcinoma and 130 invasive breast cancer tissues was evaluated using CISH. miR-320a expression levels were found to be lower in invasive breast cancer when compared with *in situ* breast carcinoma, which suggested that miR-320a may be associated with the progression of breast cancer. However, no significant association was identified between *in situ* breast carcinoma and invasive breast cancer without lymph node metastasis. These observations indicated that miR-320a may be involved in the later stages of cancer progression rather than primary tumor formation.

Breast cancer is a complex, heterogeneous disease and, thus, an urgent requirement remains to identify more effective biomarkers to predict patient prognosis. Over the past decade, an increasing number of biological parameters have been identified as being involved in the prognosis of breast cancer (23). ER is important in the carcinogenic process, and acts as a powerful prognostic factor for breast cancer patients (24). PR, an estrogen-regulated gene, exhibits a function in the ER pathway (18), and the presence of PR is associated with a lower frequency of metastasis. HER-2 is a member of the epidermal growth factor receptor (EGFR) family (25), which has also been used to predict the prognosis of breast cancer (26,27). However, according to the results of the current study, ER, PR and HER-2 exhibited no prognostic value for breast cancer patients, which was consistent with the results of previous studies (18,28,29). These observations, however, appeared to be inconsistent with those that suggest ER, PR and HER-2 may predict clinical outcome for breast cancer patients. The reason for this discrepancy is unclear and may be due to sample size and regional differences. Furthermore, this inconsistency demonstrates the complexity and heterogeneity of breast cancer. There is an urgent requirement for the identification of more effective biomarkers to predict patient prognosis.

Considering that miRNAs are only 18–24 nucleotides in length, they are robustly stable in formalin-fixed parraffin-embedded tissues. Previous studies have suggested that introducing miRNAs as biomarkers into clinical practice is beneficial, as miRNA data provides more reliable results than mRNA profiles (30,31). miR-150 may be considered as a potential prognosis biomarker in colorectal cancer therapy outcome (32). The overexpression of miR-21 predicts limited survival in patients with node-negative disease (19). In the present study, following the analysis of miR-320a expression in 130 invasive breast cancers, the correlation between miR-320a and patient prognosis was determined. The results showed that miR-320a had a potential function in predicting poor outcomes for invasive breast cancer patients. This result was confirmed using univariate and multivariate survival analyses, whereby low miR-320a expression was found to be an independent prognostic predictor of poor survival. In addition, miR-320a showed improved discrimination when compared with ER, PR and HER-2, by using an ROC curve (the ROC areas under the curve for miR-320a, ER, PR and HER-2 were 0.710, 0.517, 0.549 and 0.574, respectively). This result clearly suggests that miR-320a is a more reliable predictor for invasive breast cancer prognosis than commonly used biomarkers.

In addition to these results, the prognostic value of miR-320a expression in invasive breast cancer subgroups classified by lymph node metastasis status and clinical TNM stage were also analyzed. The results revealed that low miR-320a expression was associated with a poor prognosis in patients with clinical TNM stage III–IV and lymph node metastasis. Thus, it may be hypothesized that miR-320a may have increased prognostic value for advanced-stage invasive breast cancer. However, the subgroups of patients in this study were small and, therefore, a large study to confirm these data is necessary.

In conclusion, the results of this study indicated that miR-320a expression is significantly associated with the progression and prognosis of invasive breast cancer. Notably, miR-320a appears to be a better prognostic biomarker for invasive breast cancer than commonly used prognostic parameters. Further studies are required to investigate the function and the detailed mechanism of miR-320a in breast cancer.

## Figures and Tables

**Table I:** Clinicopathological characteristics and follow-up data of 130 invasive breast cancer patients.

Characteristics	Number of patients/total number (%)
Age (years)^a^	55.7 (34–87)
Histological type	
Invasive ductal	102/130 (78.5)
Lobular	15/130 (11.5)
Medullary	8/130 (6.2)
Mucinous	5/130 (3.8)
Histological grade	
Low (I)	14/130 (10.8)
Intermediate (II)	93/130 (71.5)
High (III)	23/130 (17.7)
Tumor size (cm)	
≤3.5	79/130 (60.8)
>3.5	51/130 (39.2)
Lymph node metastasis	
0	71/130 (54.6)
1–2	32/130 (24.6)
>2	27/130 (20.8)
Distant metastasis	
Yes	12/130 (9.2)
No	118/130 (90.8)
Clinical TNM stage	
I	44/130 (33.8)
II	54/130 (41.6)
III–IV	32/130 (24.6)
Estrogen receptor	
−	61/130 (46.9)
+	69/130 (53.1)
Progesterone receptor	
−	75/130 (57.7)
+	55/130 (42.3)
C-erbB-2 expression	
−	50/130 (38.5)
+	80/130 (61.5)
Menopause	
No	50/130 (38.5)
Yes	80/130 (61.5)
Alive with cancer	103/130 (79.2)
Succumbed to cancer	27/130 (20.8)

a Median (range).

TNM, tumor lymph-node metastasis.

**Table II:** Correlation between miR-320a expression and clinicopathological characteristics in invasive breast cancer (n=130).

	miR-320a expression		
		
Characteristics	High expression, n (%)	Low expression, n (%)	P-value
Age (years)			0.164
<45	9 (60)	6 (40)	
45–55	23 (38)	38 (62)	
>55	28 (52)	26 (48)	
Tumor size (cm)			0.046
≤2.5	42 (53)	37 (47)	
>2.5	18 (35)	33 (65)	
Lymph node metastasis			<0.001
0	46 (65)	25 (35)	
1–2	10 (31)	22 (69)	
>2	4 (15)	23 (85)	
Distant metastasis			0.006
No	59 (50)	59 (50)	
Yes	1 (8.3)	11 (91.7)	
Histological grade			0.745
Low (I)	7 (50)	7 (50)	
Intermediate (II)	44 (47)	49 (53)	
High (III)	9 (39)	14 (61)	
Clinical TNM stage			<0.001
I	28 (64)	16 (36)	
II	27 (50)	27 (50)	
III–IV	5 (16)	27 (84)	
Estrogen receptor			0.684
−	27 (44)	34 (56)	
+	33 (48)	36 (52)	
Progesterone receptor			0.352
−	32 (43)	43 (57)	
+	28 (51)	27 (49)	
HER-2 expression			0.290
−	26 (52)	24 (48)	
+	34 (42.5)	46 (57.5)	
Menopause			0.697
No	22 (44)	28 (56)	
Yes	38 (47.5)	42 (52.5)	

TNM, tumor lymph-node metastasis; HER-2, human epidermal growth factor-2.

**Table III:** Univariate and multivariate analysis for overall survival in invasive breast cancer patients (n=130).

		Univariate analysis	Multivariate analysis		
			
Features	Subset	P-value	HR	95% CI	P-value
Age		0.587			
Menopause	Yes/No	0.020	0.660	0.261–1.672	0.381
Chemotherapy		0.900			
Tumor size	≤2.5/>2.5	0.230			
Lymph node metastasis	N0/N1–2, >N2	0.076			
Clinical TNM stage	I/II, III, IV	0.010	4.434	2.308–8.522	<0.001
Histological grade	I/II, III	0.977			
Estrogen receptor	−/+	0.802			
Progesterone receptor	−/+	0.445			
HER-2 expression	−/+	0.650			
Distant metastasis	Yes/No	0.001	0.381	0.130–1.119	0.079
miR-320a	High/Low	0.015	0.221	0.050–0.979	0.047

TNM, tumor lymph-node metastasis; HER-2, human epidermal growth factor-2; HR, hazard ratio; CI, confidence interval.
